# A Rare Case of Invasive Aspergillosis of the Pituitary Gland in a Young Immunocompetent Host: Diagnostic Pitfalls and Postoperative Complications

**DOI:** 10.7759/cureus.65470

**Published:** 2024-07-26

**Authors:** Quratulain Tariq, Irfan Yousaf, Taha Ahmad, Qudsia Ahmad, Saad Bin Anis

**Affiliations:** 1 Neurosurgery, Shaukat Khanum Memorial Cancer Hospital & Research Centre, Lahore, PAK; 2 General Surgery, Shaukat Khanum Memorial Cancer Hospital & Research Centre, Lahore, PAK; 3 Surgery, Quetta Institute of Medical Sciences, Lahore, PAK

**Keywords:** para sellar extension, sellar and para sellar tumors, immunocompetent patients, pituitary gland, invasive aspergillosis

## Abstract

Invasive aspergillosis (IA) is a rare occurrence, but it should be considered in cases involving pituitary or sellar masses. Here, we present a unique case report of IA affecting the sellar region with para-sellar extension and bilateral carotid artery impingement, notably with minimal involvement of paranasal sinuses. The patient, a 16-year-old immunocompetent female from a developing country, presented without any comorbidities or classic risk factors typically associated with IA. Her initial symptoms included headaches, diplopia, and nausea. Clinically and radiologically, the patient was initially diagnosed with either craniopharyngioma or pituitary macroadenoma. Hormonal studies revealed panhypopituitarism. Previous reports of IA have not described cases with these specific presentations, particularly in this age group and immune status.

## Introduction

Aspergillosis is a fungal infection resulting from the inhalation of *Aspergillus *conidia, commonly observed in immunocompromised patients, with both invasive and noninvasive forms of the disease [1]. The incidence of aspergillosis has significantly increased due to advancements in chemotherapeutic agents [2]. These infections pose a challenge for treatment due to the limited ability of antifungal drugs to penetrate the blood-brain barrier [3]. Several factors contribute to the development of such infections, including recipients of hematopoietic stem cell transplantation (HSCT), prolonged neutropenia post-chemotherapy, long-term corticosteroid use, low CD4 counts, and extended stays in the ICU [4]. However, the case under review does not meet any of these criteria, making it a rare presentation.

## Case presentation

A 16-year-old unmarried female presented with diffuse headaches, predominantly on the right side, with a pain score of 2 out of 10, along with diplopia and nausea that had persisted for the past two months. She had no underlying medical conditions, no history of drug or substance abuse, and no prior medical or surgical interventions. Her menarche occurred at age 12, and she had a regular menstrual cycle. A previous brain MRI conducted at an external facility revealed a pituitary lesion with suprasellar extension, which exerted pressure on the optic chiasm and was initially diagnosed as either a pituitary macroadenoma or craniopharyngioma.

Ten days later, she presented to the emergency department with worsening symptoms, including right-sided visual loss, severe headaches (pain score of 8), and loss of appetite. At that time, her weight was 53 kg. Ophthalmological examination revealed bilateral ptosis, dilated pupils, unresponsiveness of the right eye to light, and temporal hemianopia in the left eye. A detailed history and physical examination were unremarkable for any systemic symptoms. She was graded as minimally active on the Lansky scale.

**Figure 1:**
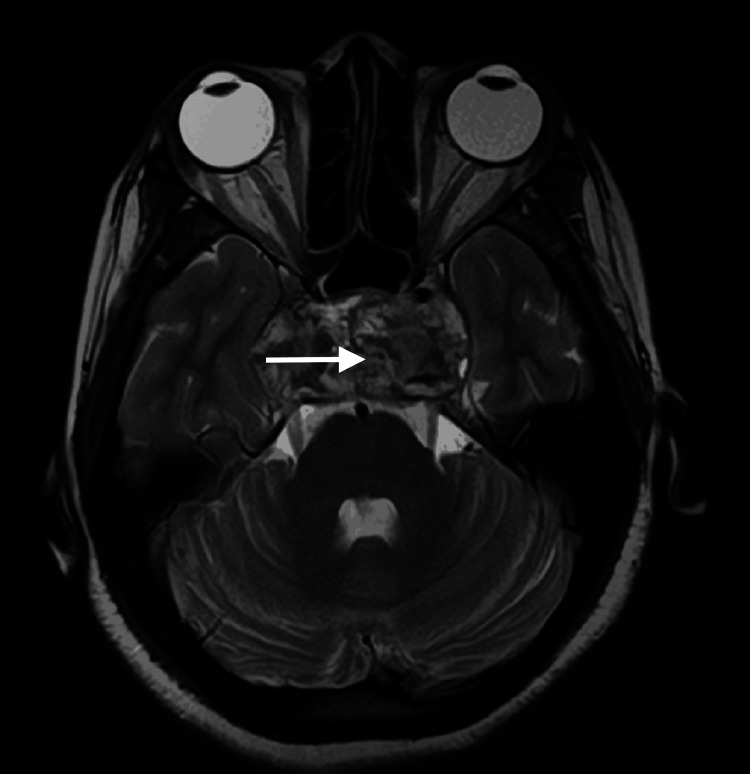
Invasive sellar and suprasellar mass in axial view (white arrow)

**Figure 2:**
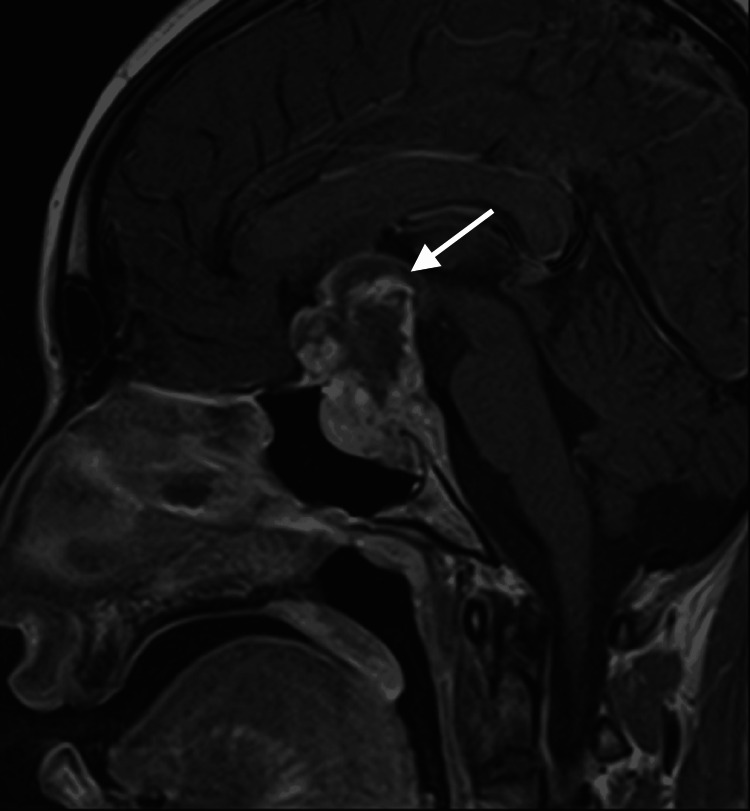
Invasive sellar and suprasellar mass in sagittal view (white arrow)

Subsequent MRI reevaluations (Figures 1, 2) at our institution showed a lobulated, heterogeneous lesion in the sellar and suprasellar regions. This lesion replaced normal pituitary tissue, caused mild elevation of the base of the third ventricle, and exerted para-sellar impingement on the internal carotid arteries bilaterally.

Laboratory investigations revealed low cortisol levels, and appropriate management was initiated. A follow-up MRI demonstrated a heterogeneous, patchy, cystic, and solid enhancing lesion involving the sellar floor, cavernous sinus, internal carotid arteries, and optic tract, with evidence of a lacunar infarct in the right internal capsule. The possibility of glioma or pituitary carcinoma was considered, and a tumor board recommended a biopsy, which was scheduled for six days later.

**Figure 3:**
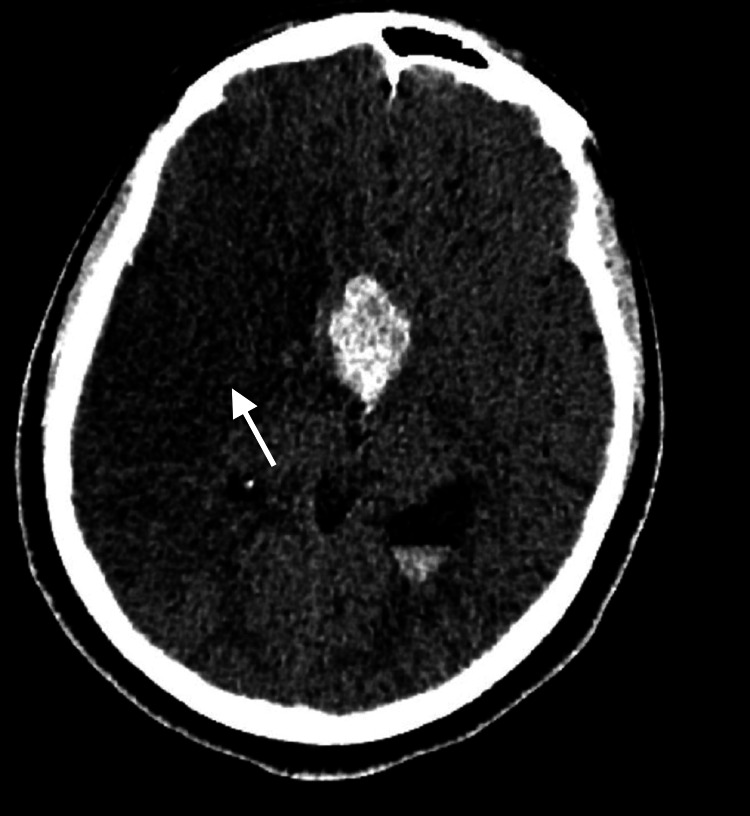
Post-biopsy right MCA territory infarct (white arrow), intralesional bleed, and intraventricular hemorrhage MCA, middle cerebral artery

Upon admission for the biopsy, the patient exhibited further deterioration of visual acuity in the left eye, bilateral complete ophthalmoplegia, and pupil dilation. Due to language barriers, perimetry could not be conducted. An endoscopic transsphenoidal biopsy of the pituitary tumor was performed, revealing pale white, glistening tissue with fine serpiginous vessels eroding the sellar floor and extending into the sphenoid sinus. Following the procedure, the patient developed distress, with decreased urinary output and electrolyte imbalances. Respiratory acidosis and decreased consciousness necessitated intubation. Imaging (Figure 3) revealed a large infarct in the right parietofrontal region and basal ganglia, along with mass effects on the sellar and suprasellar regions and intraventricular hemorrhage.

Histopathological examination revealed septate fungal hyphae with tissue invasion within necrotic debris, consistent with aspergillosis. Voriconazole treatment was initiated, but the patient’s condition deteriorated further. Despite all efforts, this led to brainstem death, refractory diabetes insipidus, and ultimately death.

## Discussion

The presented case of invasive aspergillosis (IA) in a 16-year-old immunocompetent female from Afghanistan underscores the importance of considering rare fungal infections in the differential diagnosis of sellar and suprasellar lesions. This case is particularly noteworthy due to the absence of classical risk factors associated with aspergillosis, such as HSCT, prolonged neutropenia, or chronic corticosteroid therapy. According to the Infectious Diseases Society of America, a high level of clinical suspicion in the presence of risk factors is required to start antifungal therapy before a histopathology report is received [5]. For an immunocompetent patient, an extension from a lesion of invasive *Aspergillus* sinusitis is the most probable cause of IA, but no such history was present.

The initial clinical presentation of diffuse headaches, diplopia, and nausea, along with radiological findings suggestive of a pituitary macroadenoma or craniopharyngioma, posed a diagnostic challenge [6]. Despite the utilization of brain MRI, the definitive diagnosis was delayed, highlighting the complexity of differentiating between neoplastic and infectious etiologies in sellar lesions [7].

The delayed initiation of appropriate antifungal therapy due to diagnostic uncertainties likely contributed to the poor outcome in this case. Despite the subsequent administration of voriconazole upon histopathological confirmation of aspergillosis, the patient’s clinical deterioration was irreversible, ultimately resulting in brain stem death.

Further complicating the diagnostic process were the evolving clinical manifestations, including worsening visual acuity, complete ophthalmoplegia, and refractory diabetes insipidus. These neurological sequelae, coupled with radiological evidence of infarction and intraventricular hemorrhage, underscore the aggressive nature of IA and its potential to lead to life-threatening complications.

This case emphasizes the importance of maintaining a high index of suspicion for fungal infections, even in immunocompetent individuals presenting with atypical clinical features [8]. Early recognition and prompt initiation of antifungal therapy are crucial in improving patient outcomes and mitigating the risk of irreversible neurological sequelae associated with IA [9]. Further research is warranted to elucidate the optimal diagnostic and therapeutic strategies for managing rare fungal infections in the sellar and suprasellar regions, particularly in resource-limited settings.

## Conclusions

Research on the etiology and epidemiology of IA in immunocompetent hosts will help in hinting toward a diagnosis and early initiation of treatment in the absence of typical symptoms and therefore prevention of increased morbidity and mortality. Efforts should be made for the proper allocation of resources and bridging treatment delays systematically.
